# N6-methyladenosine of *TRIM27* enhances the stem cell-type phenotype of cisplatin-resistant colorectal cancer cells

**DOI:** 10.1016/j.bbrep.2023.101572

**Published:** 2023-11-09

**Authors:** Jun-qiong Zheng, Ying Zhan, Wen-jing Huang, Zhi-yong Chen, Wei-hao Wu

**Affiliations:** Department of Medical Oncology, Longyan First Hospital Affiliated to Fujian Medical University, Longyan, Fujian, China

**Keywords:** Colorectal cancer, Cancer stem cell, m^6^A, *TRIM27*, *YTHDF1*

## Abstract

Colorectal cancer (CRC), classified as a lethal form of cancer, substantially threatens human well-being. Cancer stem cells (CSCs) reflect subsets for cancerous cells having basic stem-cell type properties, being significantly involved in the development of chemoresistance and tumor relapsing. The aberrant *TRIM27* expression in various types of cancer indicates its potential involvement in cancer growth and progression. The current understanding of the *TRIM27* involvement in CRC remains limited. In current study indicated that *TRIM27* can potentially promote CSC-type phenotype of Cisplatin (DDP)-resistant CRC cells. *YTHDF1* recruitment onto m^6^A-amended *TRIM27* was crucial for facilitating the *TRIM27* translating process in DDP-resistant CRC cells. The present research proposes that *TRIM27* exhibits an oncogenic role by enhancing the CSC-type properties in DDP-resistant CRC *via* the m^6^A-modified pathway. The potential therapy for combating the relapse of CRC may include *TRIM27* and *YTHDF1*, as they have been found to have significant roles in promoting CSC-type phenotypic characteristics.

## Highlights

• *TRIM27* protein level was increased in cisplatin (DDP)-resistant CRC.
• *TRIM27* enhanced the CSCs-type characteristics in DDP-resistant CRC.
• *TRIM27* translation was enhanced in DDP-resistant CRC cells.
• *YTHDF1* interaction with the *TRIM2*7 mRNA and enhances its protein expression in DDP-resistant CRC cells.

## 1 Introduction

Colorectal cancer (CRC) is a prevalent malignancy responsible for significant cancer-related deaths globally, with an elevated mortality rate and adverse prognosis in cancer patients [1]. Cisplatin, an antineoplastic agent derived from platinum, has extensively demonstrated its efficacy in treating various malignancies [2]. The clinical use of cisplatin results in notable improvements in the prognosis and survival rates of cancer patients. However, its widespread implementation is hindered by substantial challenges, including the emergence of drug resistance and the occurrence of significant side effects [2]. The evidence consistently demonstrates that CRC exhibits a notable heterogeneity and covers a small fraction of tumor-inducing cells called cancer stem cells (CSCs). They perform a crucial function in replenishing chemotherapy-resistant CRC, thereby facilitating continuous growth and relapse [3]. Despite the effectiveness of conventional therapies in eliminating primary tumors, the presence of CSCs within the malignant tissue can contribute to recurrence due to their inherent CSC characteristics, including self-renewal, differentiation, and tumorigenesis. Exploration of tumor properties resembling CSCs can be effectively conducted *via* induction of subcutaneous tumor development in BALB/c nude mice [4]. Hence, targeting CSCs presents significant therapeutic prospects in addressing the advancement and relapse of CRC. Therefore, comprehensive cellular attributes while self-regenerating capacity by CRC CSCs offers valuable opportunities for developing treatment strategies for CRC.

The tripartite motif (*TRIM*) protein family is recognized by the existence of three distinct domains: a coiled-coil region, a domain comprising B-box domains, and a RING finger domain. Notably, the C-terminal region of *TRIM* proteins exhibits significant variability among different family members [5]. *TRIM27* protein, a member of the *TRIM* protein family, is present in many human organs [6]. In recent investigations, the oncogenic function of *TRIM2*7 has been elucidated in diverse malignancies, including lung carcinoma, endometrial carcinoma, and breast carcinoma [[7], [8], [9]]. The presented evidence demonstrates a consistent association between the *TRIM27* expression and the natural progression of cancer. These findings suggest that this gene and its encoded products play a remarkable role in cancer development.

Genes *METTL3, METTL14,* and *WTAP* have been identified as key players in m^6^A methylation that can be reversed through m^6^A erasers, specifically the fat-mass and obesity-linked protein (FTO) together with AlkB homolog 5 (AlKBH5) [[10], [11], [12]]. YTH domain family (*YTHDF*) together with heterogeneous nuclear ribonucleoproteins (hnRNPs) serve as m^6^A readers, enabling the recognition and subsequent regulation of downstream molecular mechanisms [[13], [14], [15]]. *YTHDF1* is a protein that comprises the *YT521*-B homology (YTH) domain. The recognition of m^6^A by this protein is facilitated by the conserved YTH domain, enabling it to modulate the functions of genes at the stage of post-transcription. This study has shown that the *TRIM27* protein level was elevated in both CRC tissues and cells, depending on the m^6^A*-YTHDF1* pathway. Overall findings indicate that *TRIM27* is essential in maintaining CRC CSC-type characteristics.

## 2 Materials and methods

### 2.1 Cell culture

The RPMI-1640 (Life Technologies, USA) medium was used to culture the human CRC cell lines (HCT116; SW620) along with their cisplatin-resistant equivalents (HCT116/DDP; SW620/DDP), and subsequently maintained with 5 % CO2 at 37 °C. Penicillin, Streptomycin (100 U/mL), and fetal bovine serum (10 % FBS) (Sigma, USA) were added to enrich the medium for growth.

### 2.2 CRISPR-Cas9 cell line formation

A lentivirus infection experiment was conducted to achieve stable gene knockout. The lentivirus used in this study was procured from Nanjing Jikai Gene Corporation. Approximately 100,000 cells were cultured within six-well plates overnight. The cell culture medium was supplemented with viral solutions based on the multiplicity of infection (MOI) to induce infection. Following an 8 h incubation, the medium was refreshed with a normal culture medium supplemented through 1 μg/mL Puromycin (two-day exposure). Cultures were propagated, expended/validated *via* Western blotting (WB) analysis.

### 2.3 Formation of spheroids

Cultures undergoing logarithmic expansion were trypsinized/maintained within 10 % serum-harboring medium. Cells were resuspended within serum-free medium after centrifugation at 500 rpm/min. They were grown onto ultralow-attachment six-well plate (Corning™, USA), maintained in a culture medium consisting of 3 mL DMEM/F12, 20 mg/ml human recombinant epidermal growth factor (Sigma Aldrich™, USA), 5 μg/mL insulin (Sigma-Aldrich™), and 2 % B27 (Invitrogen™, USA). Culture media were replenished at 72 h intervals. Cell imaging was performed *via* BioTek Cytation 5® cellular imaging multimode reader following a cell seeding of 7∼10 days. Only spheroids with a diameter greater than 50 μm were quantified.

### 2.4 m^6^A RNA immunoprecipitation(RIP) assay

Total RNA was extracted from the respective CRC cells using TRIzol reagent (Invitrogen, Waltham, MA, USA). RIP was conducted with the Magna RIP RNA-Binding Protein Immunoprecipitation Kit (#17–10499, Merck Millipore™, USA) according to the manufacturer's instructions. Enrichment of m^6^A was analyzed using RT-PCR analysis.

### 2.5 Clinical tissues

In this study, 12 patients have undergone surgical procedures at Longyan First Hospital, Affiliated with Fujian Medical University. Before the surgery, they were not exposed to chemotherapy or radiotherapy. 6 patients had DDP-resistant (defined as tumour recurrence within six months after R0 excision during DDP-based therapy). 6 patients had DDP-sensitive (defined as no tumour recurrence during DDPbased therapy). The harvested tissues were placed in liquid nitrogen and then cryopreserved at −80 °C for subsequent examinations. Cases were segregated within separate distinct cohorts. The project received approval from the Institutional Review Board (IRB) of Longyan First Hospital, affiliated with Fujian Medical University. All procedures involving human participants in this study were conducted in accordance with the Declaration of Helsinki (as revised in 2013), and all patients or their respective families were provided with the study's rationale and obtained their consent prior to the participation.

### 2.6 m^6^A determination

Overall m^6^A RNA presence changes were quantified *via* EpiQuik m^6^A RNA Methylation Quantification Kit (Colorimetric) (#P-9005, Epigentek, Farmingdale, NY) in accordance with established guidelines provided by the manufacturer. The subsequent analysis required 200 ng of RNA from both tested CRC tissues and cell lines.

### 2.7 Western blotting

Cell lysates or tissue extracts were prepared following the previously established protocol [15]. SDS-PAGE was performed to isolate all the proteins and transfer them onto 0.45 μm PVDF membranes (Millipore, IPFL85R). After blocking the PVDF membranes with TBS-T (5 % skim milk), followed by primary and secondary antibodies (1 h at RT), the proteins were visualized in accordance with the manufacturer's guidance *via* SuperSignal West Pico PLUS (Invitrogen,34,580).

### 2.8 Real-time(RT)-PCR

Total RNA was isolated from CRC tissues or cells using Trizol reagent (Invitrogen, Waltham, MA, USA), and then RNA was used to perform reverse transcription with a Transcriptor First Strand cDNA Synthesis kit (Roche, Basel, Switzerland). The acquired cDNAs were used as templates for real-time PCR analysis using SYBR Green PCR Master Mix (Qiagen, Valencia, CA, USA). The relative mRNA levels were calculated using the 2^−ΔΔCt^ method, with the levels normalized to GAPDH mRNA.

### 2.9 RNA lifetime assays

Cells were treated according to our experimental design. Actinomycin D (Sigma) was added at a concentration of 5 mg/mL. Then, at the indicated times, cells were lysed and the total RNA was extracted (Qiagen). RNA quantities were determined through q-PCR analysis.

### 2.10 Statistical analysis

One-way ANOVA analyzed the significance level of the disparity in all experiments, followed by post-hoc Dunnett's assessment. The log-rank assessment determined the *p-*value for Immunohistochemical and microarray analyses. The cutoff for the significance level was *p* < 0.05. All experiments were executed in triplicates, and data were stated as the mean ± SD.

## 3 Results

### 3.1 *TRIM27* protein was increased in cisplatin (DDP)-resistant CRC

**Fig. 1:**
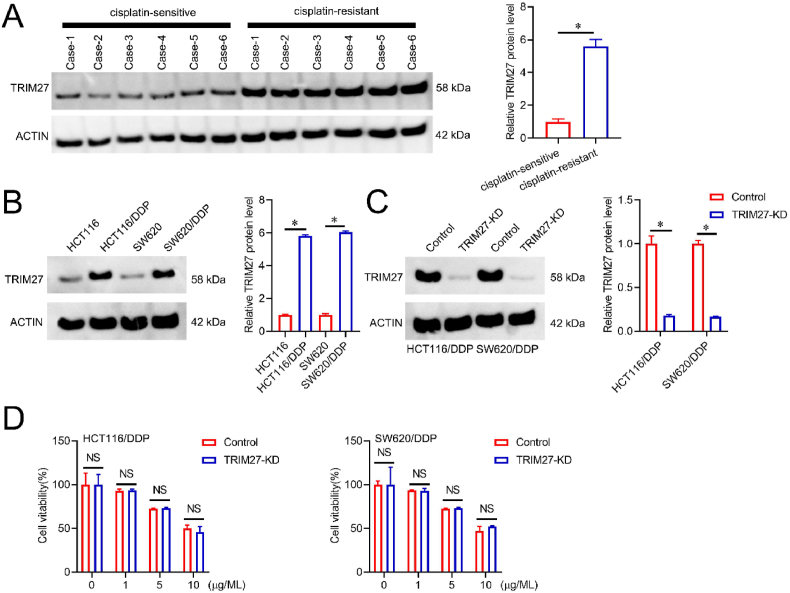
Elevation of *TRIM27* protein in cisplatin-resistant CRC A. Representatives immunoblot (left) and quantification (right) of TRIM27 protein level expression in DDP-resistant CRC tissues. **B**. Representatives immunoblot (left) and densitometric analysis (right) of protein expression of TRIM27 in HCT116 and SW620 and DDP-resistant cells. **C**. Knocking down the TRIM27 expression *via* CRISPR-Cas9, and the efficacy was validated by Western blotting (left) and densitometric analysis (right). **D**. CCK8 analysis was used to evaluate cell viability in HCT116/DDP or SW620/DDP or TRIM27 (KD) cells treated with different concentrations of DDP for 48 h, *p < 0.05; N.S.not significant.

We firstly analyzed *TRIM27* protein level in 6 cisplatin-sensitive and resistant CRC tissues. *TRIM27* expression was substantially increased in DDP-resistant CRC tissues (N = 6) compared to DDP-sensitive CRC tissues (N = 6) (Fig. 1A). Meanwhile, we also found that *TRIM27* protein level enhanced in the DDP-resistant HCT116 (HCT116/DDP) and SW620 (SW620/DDP) cells in comparison with control cells (Fig. 1B). The system of CRISPR/Cas9 was utilized to knockdown the expression of *TRIM27* in the HCT116/DDP and SW620/DDP cell lines (Fig. 1C); nevertheless, *TRIM27* knockdown failed to significantly affect the sensitivity of HCT116/DDP or SW620/DDP cells towards DDP treatment (Fig. 1D). These findings demonstrated that *TRIM27* may not be necessary or implicated in DDP-resistant CRC, even though it was elevated in DDP-resistant CRC.

### 3.2 Role of *TRIM27* in maintaining the CSCs-type characteristics in DDP-resistant CRC

**Fig. 2:**
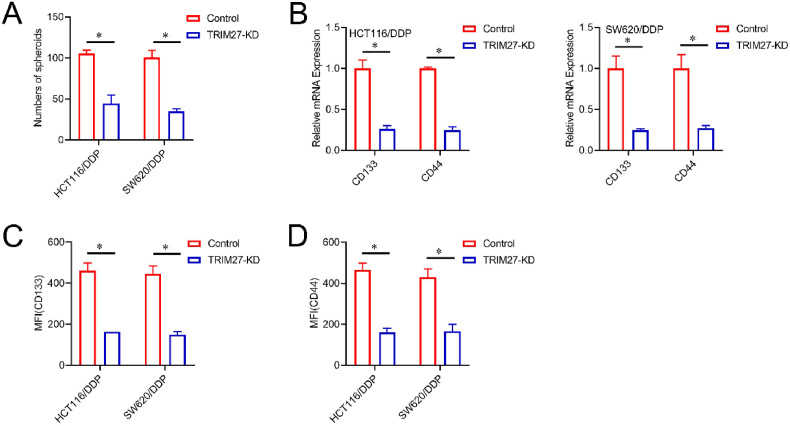
Role of *TRIM27* in maintaining CSCs-type characteristics of DDP-resistant CRC cells.**A**.Spheroids formation and quantification from control or TRIM27-KD HCT116/DDP and SW620/DDP cells, **B**. Analysis of CD133 and CD44 expressions *via* RT-PCR. **C-D**. Flow cytometry analysis of CD133 (C) and CD44 expressions (D), and data were recorded *via* MFI (mean fluorescence intensity).*p < 0.05.

Spheroid formation assay exhibited that *TRIM27* knockdown reduced the spheroid-forming capability of the DDP-resistant CRC cells (Fig. 2A and Fig. S1). RT-PCR analysis exhibited a substantial decrease in the expression of CRC stem-cell epitopes CD133/CD44 within HCT116/DDP or SW620/DDP cultures upon *TRIM27* knockdown(Fig. 2B). Furthermore, flow cytometry analysis validated the downregulation of cellular superficial markers; CD133/CD44 within HCT116/DDP or SW620/DDP cultures following *TRIM27* knock-down (Fig. 2C-D). These findings demonstrated that *TRIM27* enhanced the CSCs-type characteristics in DDP-resistant CRC.

### 3.3 Upregulated *TRIM27* translation in DDP-resistant CRC cells

**Fig. 3:**
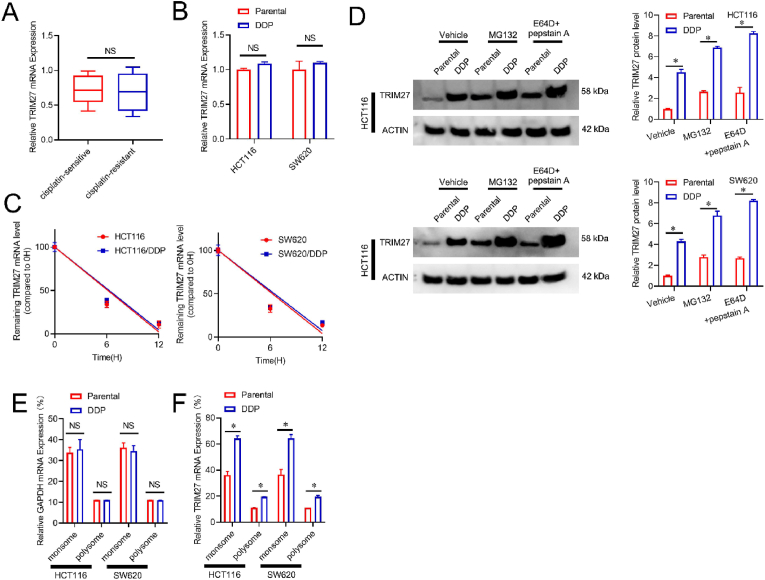
Upregulation of *TRIM27* translation in DDP-resistant CRC cells. A-B. Analysis of *TRIM2*7 mRNA level by real-time PCR within CRC tissue (A) or cellular cultures (B). **C**. Shortened RNA lifetime of TRIM27 mRNA in indicated CRC cell or CRC-DDP cells. **D**. Respective immunoblots (left) and quantification (right) of TRIM27 levels in HCT116 or SW620 cells treated with vehicle (SPECIFY VEHICLE USED), MG132 or E64D, and pepstatin A (one day). **E**. RT-PCR analysis for *TRIM2*7 mRNA distribution, **F**. Real-time PCR analysis for GAPDH mRNA distribution.*p < 0.05; N.S.not significant.

Following, we investigated of the molecular mechanisms responsible for the *TRIM27* upregulation in DDP-resistant CRC, and RT-PCR was executed to investigate the mRNA level of *TRIM27* in DDP-sensitive and DDP-resistant CRC tissues and across different cancer cell lines. The findings revealed an insignificant disparity between DDP-resistant and sensitive CRC cells or tissues, suggesting that the *TRIM27* expression is regulated at the translation phase (Fig. 3A-B). Furthermore, the *TRIM2*7 mRNA degradation exhibited comparable patterns in both DDP-resistant and control CRC cells (Fig. 3C). *TRIM27* protein level *was both* up-regulated *in* HCT116-control and SW620-control *cells or* HCT116-DDP and SW620-DDP *cells under* MG132 introduction, a potent proteasome regulator, or E64D/pepstatin A, lysosomal regulators*, however,* upregulated *TRIM27 in* HCT116-DDP and SW620-DDP compared to HCT116-control and SW620-control cells were unchanged under the same condition treated(MG132 or E64D/pepstatin A) (Fig. 3D). These findings demonstrate that the *TRIM2*7 mRNA and protein degradation remains unchanged in DDP-resistant CRC cells. Furthermore, *TRIM2*7 mRNA was substantially elevated on monosomes and polysomes of DDP-resistant CRC cells relative to the control cells (Fig. 3E). In comparison to the DDP-sensitive CRC cells, GAPDH transcriptomic expression remained unchanged within DDP-resistant CRC cells(Fig. 3F). Above findings suggested a potential involvement of translational activation in the up-regulated of *TRIM27* in DDP-resistant CRC cells.

### 3.4 *YTHDF1* interaction with the *TRIM2*7 mRNA and enhances its protein expression in DDP-resistant CRC cells

**Fig. 4:**
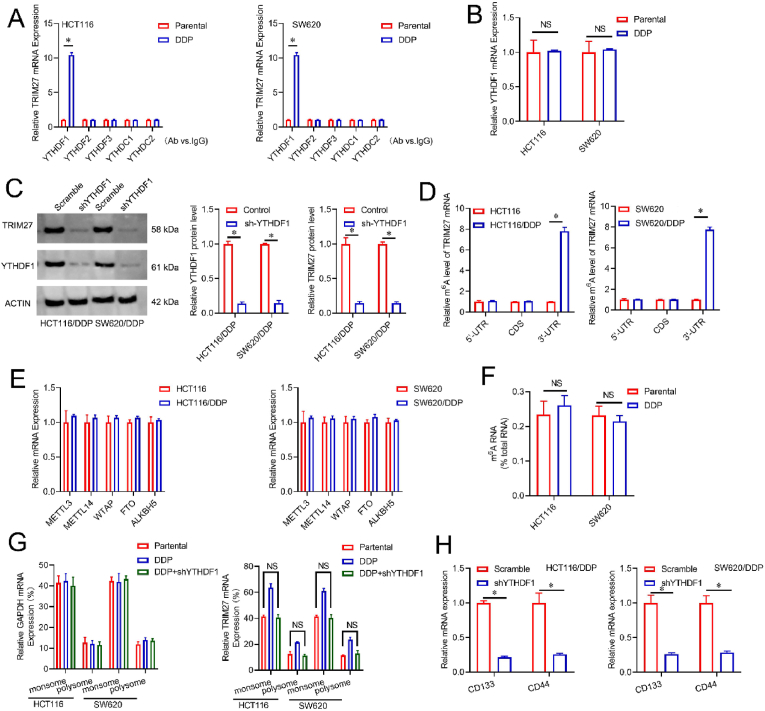
*YTHDF1* interaction with the *TRIM2*7 mRNA and enhances its protein expression in DDP-resistant CRC cells. A. RIP evaluation *via* IgG or *the YTHDF1-3, YTHDC1-2* antibodies, and RT-PCR analysis for *TRIM2*7 mRNA enrichment. **B**. Analysis of *YTHDF1* mRNA level by real-time PCR. **C**. Left panel:Knockdown *YTHDF1 via* shRNAs against *YTHDF1* (*shYTHDF1*), and respective antibodies analyzed *via* Western blot, Right panel: Duantify densitrometric analysis. **D**. m^6^A RIP-PCR analysis by IgG or an m^6^A antibody. **E**. Real-time PCR analysis for *METTL3, METTL14, WTAP, ALKBH5*, and *FTO* mRNA expression.**F**.Quantification of total m^6^A RNA levels *via* ELISA. **G**. Real-time PCR analysis for *TRIM27* and GAPDH mRNA distribution.**H.** Analysis of stem cell markers *via* RT-PCR. *p < 0.05; N.S.not significant. *p < 0.05; N.S. not significant.

The m^6^A amending is identified by specific readers in translating target mRNAs(16). RIP analysis revealed that the *TRIM2*7 mRNA level interaction with YTHDF1 was increased in HCT116/DDP and SW620/DDP cell lines compared to the control cells, while the *TRIM2*7 mRNA level interaction with additional m^6^A readers was consistent in HCT116/DDP and SW620/DDP cell lines compared to the control cells (Fig. 4A). Although DDP-resistant cells expressed *YTHDF1* similarly to control cells (Fig. 4B), *YTHDF1* knocked down reduced the protein *TRIM27* level in HCT116/DDP and SW620/DDP cells (Fig. 4C). These results suggested that *YTHDF1* may promote *TRIM27* translation in DDP-resistant CRC cells by interacting with its transcript. Subsequently, an investigation was also conducted to ascertain the potential alteration of m^6^A amending in the *TRIM27* transcript. The application of MeRIP, utilizing a specific antibody targeting m^6^A amending, revealed noteworthy findings regarding the enrichment of *TRIM27* 3′UTR in HCT116/DDP and SW620/DDP cells (Fig. 4D). These outcomes indicate a potential role of *YTHDF1* in facilitating the *TRIM2*7 mRNA translation through its ability to recognize m^6^A sites on the 3′UTR of the mRNA. To investigate the potential mechanism responsible for the m^6^A upregulation in the *TRIM27* transcript, this study focused on examining the patterns of m^6^A writers or erasers. Surprisingly, this investigation revealed that the level of *METTL3, METTL14, WTAP*, *ALKBH5*, together with *FTO* did not show any notable variation when comparing control CRC cells to DDP-resistant cells (Fig. 4E). The quantification for global m^6^A RNA was conducted, revealing not notable variation between HCT116/DDP or SW620/DDP cells and their control cells (Fig. 4F). Meanwhile, *TRIM2*7 mRNA elevated on monosomes and polysomes of DDP-resistant CRC cells relative to the control cells was impaired by *YTHDF1* knocked down(Fig. 4G). Additionally, the knockdown of *YTHDF1* led to the corresponding suppression of CSCs-marker *CD133, an*d CD44 expressions (Fig. 4H). These findings demonstrated that *YTHDF1* interaction with the *TRIM2*7 mRNA and enhances its protein expression in DDP-resistant CRC cells.

## 4 Discussion

The current findings presented evidence indicating an upregulation of *TRIM27* in DDP-resistant CRC cells at the protein level, and *TRIM27* knockdown remarkably reduced the expression of CSC resembling characteristics. Knock down TRIM27 inhibited the CSCs-type characteristics in DDP-resistant CRC Overall, indicating that *TRIM27* is essential in maintaining CRC CSC-type characteristics.

The m^6^A amending is extensively recognized as a predominant occurring, widespread, and conserved amending in various RNA molecules. Its significance lies in its emerging role as a novel regulator of RNA processing, transcriptional regulation, alternative splicing, RNA stability, and translation control [[16], [17], [18], [19], [20]]. The present investigation has revealed DDP-resistant tumors exhibit comparable levels for m^6^A amending compared to the control cells. There is a noteworthy upregulation in the m^6^A amending for *TRIM27*. However, dynamic and reversible m^6^A amending is primarily facilitated by specific enzymes known as writers (*METTL3, METTL14*, and *WTAP*) and erasers (FTO and ALKBH5) [[21], [22], [23]]. No common amendings of m^6^A writers/erasers were identified within this study, which may explain the improvement of *TRIM2*7 m^6^A amending in DDP-resistant CRC. Additional investigations are warranted to explore the precise mechanism(s) responsible for enhancing m^6^A amending for *TRIM27* within DDP-resistant CRC.

The m^6^A amending remains identified through various readers, such as *YTHDF1-3, YTHDC1-2*, together with hnRNP family proteins [[24], [26], [25]]. *YTHDF1* can identify m^6^A post-transcriptional amending *via* a conserved aromatic cage upon YTH region [[27], [28], [29]]. Despite the shared ability of YTH domain-containing proteins to interact with m^6^A transcriptomic locations, exhibiting distinct target mRNA recognition while performing different roles. Case in point, the translational efficiency of the mRNAs regulated by *YTHDF1, YTHDF3*, and *YTHDC2*, their stability was reduced by *YTHDF2, YTHDF3, and YTHDC2*, while *YTHDC1* substantially involved in m^6^A mRNAs splicing and nuclear export [30]. The present investigation has revealed that there is an enhancement in the recruitment of *YTHDF1* to *TRIM2*7 mRNA within DDP-resistant CRC cells. This heightened recruitment facilitated the translation of the *TRIM27* transcript, While *TRIM27* protein expression was considerably reduced by *YTHDF1* knockdown. The existing studies indicate that m^6^A amending dysregulation is closely linked to the onset and advancement of diverse types of human cancers. Besides, m^6^A amending can act as either an oncogene or a tumor suppressor based on the specific characteristics of the cancer under consideration [31]. Henceforth, the findings from this study strongly suggest that *YTHDF1* exhibits promising effects as a viable therapeutic management of CRCs.

## 5 Conclusion

This study presented the involvement of *TRIM27* as an oncogene that promotes the CSC-type properties of CRC cells. Further, *YTHDF1*, an upstream molecule for *TRIM27*, could identify delf 3′UTR, facilitating its translation within CRC. Hence, drawing upon the observed link between *TRIM27* and the CSC-type properties in DDP-resistant CRC cells, *TRIM27* holds promise as a possible therapeutic target for treating CRC.

## Ethics approval

The Ethics Committee of Longyan First Hospital Affiliated to Fujian Medical University approved the research. All procedures involving human participants in this study were conducted in accordance with the Declaration of Helsinki (as revised in 2013), and all patients or their respective families were provided with the study's rationale and obtained their consent prior to the participation.

## Informed consent

Written informed consent was obtained from all the patients.

## Funding

None.

## Authors’ contribution

Jun-qiong Zheng, Ying Zhan, Wen-jing Huang, Zhi-yong Chen, and Wei-hao Wu contributed significantly to performing the experiments and assisted in writing the manuscript; Wei-hao Wu and Zhi-yong Chen contributed to the conception of the study. All authors read and approved the final study.

## Declaration of competing interest

The Authors declare that they have no conflict of interests.

## Data Availability

The data that has been used is confidential.

## References

[1] Li W.H., Zhang L., Wu Y.H. (2020 Apr). CDKN3 regulates cisplatin resistance to colorectal cancer through TIPE1. Eur. Rev. Med. Pharmacol. Sci..

[2] Lv Q., Xia Q., Li A., Wang Z. (2022 Mar 14). circRNA_101277 influences cisplatin resistance of colorectal cancer cells by modulating the miR-370/IL-6 Axis. Genet. Res..

[3] Wang J., Yang P., Yu T., Gao M., Liu D., Zhang J., Lu C., Chen X., Zhang X., Liu Y. (2022 Oct 24). Lactylation of PKM2 suppresses inflammatory metabolic adaptation in pro-inflammatory macrophages. Int. J. Biol. Sci..

[4] Yang J., Luo L., Zhao C., Li X., Wang Z., Zeng Z., Yang X., Zheng X., Jie H., Kang L., Li S., Liu S., Zhou C., Liu H. (2022 May 13). A positive feedback loop between inactive VHL-triggered histone lactylation and PDGFRβ signaling drives clear cell renal cell carcinoma progression. Int. J. Biol. Sci..

[5] Yu C., Rao D., Wang T., Song J., Zhang L., Huang W. (2022 Sep 19). Emerging roles of TRIM27 in cancer and other human diseases. Front. Cell Dev. Biol..

[6] Jia X., Zhao C., Zhao W. (2021 Jun 10). Emerging roles of MHC class I region-encoded E3 ubiquitin ligases in innate immunity. Front. Immunol..

[7] Yang Y., Zhu Y., Zhou S., Tang P., Xu R., Zhang Y., Wei D., Wen J., Thorne R.F., Zhang X.D., Guan J.L., Liu L., Wu M., Chen S. (2022 Jul 18). TRIM27 cooperates with STK38L to inhibit ULK1-mediated autophagy and promote tumorigenesis. EMBO J..

[8] Miao X., Xiang Y., Mao W., Chen Y., Li Q., Fan B. (2020 Feb 1). TRIM27 promotes IL-6-induced proliferation and inflammation factor production by activating STAT3 signaling in HaCaT cells. Am. J. Physiol. Cell Physiol..

[9] Wang J., Zhao D., Lei Z., Ge P., Lu Z., Chai Q., Zhang Y., Qiang L., Yu Y., Zhang X., Li B., Zhu S., Zhang L., Liu C.H. (2023 Feb). TRIM27 maintains gut homeostasis by promoting intestinal stem cell self-renewal. Cell. Mol. Immunol..

[10] Chen J., Fang Y., Xu Y., Sun H. (2022 May 16). Role of m6A amending in female infertility and reproductive system diseases. Int. J. Biol. Sci..

[11] Qin S., Mao Y., Chen X., Xiao J., Qin Y., Zhao L. (2021 Jul 22). The functional roles, cross-talk and clinical implications of m6A amending and circRNA in hepatocellular carcinoma. Int. J. Biol. Sci..

[12] Song H., Wang L., Chen D., Li F. (2020 Jan 1). The function of pre-mRNA alternative splicing in mammal spermatogenesis. Int. J. Biol. Sci..

[13] Shi J., Zhang Q., Yin X., Ye J., Gao S., Chen C., Yang Y., Wu B., Fu Y., Zhang H., Wang Z., Wang B., Zhu Y., Wu H., Yao Y., Xu G., Wang Q., Wang S., Zhang W. (2023 Jan 1). Stabilization of IGF2BP1 by USP10 promotes breast cancer metastasis via CPT1A in an m6A-dependent manner. Int. J. Biol. Sci..

[14] Xu Y., Zhou J., Li L., Yang W., Zhang Z., Zhang K., Ma K., Xie H., Zhang Z., Cai L., Gong Y., Gong K. (2022 Oct 3). FTO-mediated autophagy promotes progression of clear cell renal cell carcinoma via regulating SIK2 mRNA stability. Int. J. Biol. Sci..

[15] Li Z., Yang H.Y., Dai X.Y., Zhang X., Huang Y.Z., Shi L., Wei J.F., Ding Q. (2021 Mar 15). CircMETTL3, upregulated in a m6A-dependent manner, promotes breast cancer progression. Int. J. Biol. Sci..

[16] Sun C.Y., Cao D., Du B.B., Chen C.W., Liu D. (2022 Mar 28). The role of Insulin-type growth factor 2 mRNA-binding proteins (IGF2BPs) as m6A readers in cancer. Int. J. Biol. Sci..

[17] Shi K., Yang S., Chen C., Shao B., Guo Y., Wu X., Zhao L., Yang X., Zhang Q., Yuan W., Sun Z. (2022 Apr 24). RNA methylation-mediated LINC01559 suppresses colorectal cancer progression by regulating the miR-106b-5p/PTEN axis. Int. J. Biol. Sci..

[18] Wu Q., Zhang H., Yang D., Min Q., Wang Y., Zhang W., Zhan Q. (2022 Jul 18). The m6A-induced lncRNA CASC8 promotes proliferation and chemoresistance via upregulation of hnRNPL in esophageal squamous cell carcinoma. Int. J. Biol. Sci..

[19] Li P., Shi Y., Gao D., Xu H., Zou Y., Wang Z., Li W. (2022 Oct 18). ELK1-mediated YTHDF1 drives prostate cancer progression by facilitating the translation of Polo-type kinase 1 in an m6A dependent manner. Int. J. Biol. Sci..

[20] Hu Z., Li Y., Yuan W., Jin L., Leung W.K., Zhang C., Yang Y. (2022 Sep 11). N6-methyladenosine of Socs1 modulates macrophage inflammatory response in different stiffness environments. Int. J. Biol. Sci..

[21] Li H., Zhong Y., Cao G., Shi H., Liu Y., Li L., Yin P., Chen J., Xiao Z., Du B. (2022 May 1). METTL3 promotes cell cycle progression via m6A/YTHDF1-dependent regulation of CDC25B translation. Int. J. Biol. Sci..

[22] Mao Y., Jiang F., Xu X.J., Zhou L.B., Jin R., Zhuang L.L., Juan C.X., Zhou G.P. (2023 Jan 1). Inhibition of IGF2BP1 attenuates renal injury and inflammation by alleviating m6A amendings and E2F1/MIF pathway. Int. J. Biol. Sci..

[23] Yu T., Yao L., Yin H., Teng Y., Hong M., Wu Q. (2022 Mar 6). ALKBH5 promotes multiple myeloma tumorigenicity through inducing m6A-demethylation of SAV1 mRNA and myeloma stem cell phenotype. Int. J. Biol. Sci..

[24] Oerum S., Meynier V., Catala M., Tisné C. (2021 Jul 21). A comprehensive review of m6A/m6Am RNA methyltransferase structures. Nucleic Acids Res..

[25] Qin Y., Li L., Luo E., Hou J., Yan G., Wang D., Qiao Y., Tang C. (2020 Dec). Role of m6A RNA methylation in cardiovascular disease (Review). Int. J. Mol. Med..

[26] An Y., Duan H. (2022 Jan 12). The role of m6A RNA methylation in cancer metabolism. Mol. Cancer.

[27] Sun T., Wu R., Ming L. (2019 Apr). The role of m6A RNA methylation in cancer. Biomed. Pharmacother..

[28] Deng L.J., Deng W.Q., Fan S.R., Chen M.F., Qi M., Lyu W.Y., Qi Q., Tiwari A.K., Chen J.X., Zhang D.M., Chen Z.S. (2022 Feb 14). m6A amending: recent advances, anticancer targeted drug discovery and beyond. Mol. Cancer.

[29] Liu Z.X., Li L.M., Sun H.L., Liu S.M. (2018 Jul 13). Link between m6A amending and cancers. Front. Bioeng. Biotechnol..

[30] Fang Z., Mei W., Qu C., Lu J., Shang L., Cao F., Li F. (2022 Aug 9). Role of m6A writers, erasers and readers in cancer. Exp. Hematol. Oncol..

[31] Ma S., Chen C., Ji X., Liu J., Zhou Q., Wang G., Yuan W., Kan Q., Sun Z. (2019 Nov 22). The interplay between m6A RNA methylation and noncoding RNA in cancer. J. Hematol. Oncol..

