# Supplementary material for: N6-methyladenosine of TRIM27 enhances the stem cell-type phenotype of cisplatin-resistant colorectal cancer cells
Source: Biochem Biophys Rep. 2023 Nov 9;36:101572. doi: 10.1016/j.bbrep.2023.101572 (PMC10658205; doi:10.1016/j.bbrep.2023.101572)
Supplement: Multimedia component 1 [file mmc1.docx]

**N6-methyladenosine of *TRIM27* enhances the stem cell-type phenotype of cisplatin-resistant colorectal cancer cells**

Jun-qiong Zheng, Ying Zhan, Wen-jing Huang, Zhi-yong Chen#, Wei-hao Wu#

Department of Medical Oncology, Longyan First Hospital Affiliated to Fujian Medical University, Longyan, Fujian, China

corresponding authors:

Zhi-yong Chen.

Email: 820438840@qq.com;

Wei-hao Wu,

Email: [714451648@qq.com &](mailto:714451648@qq.com%20&) jiayou061516@163.com

**
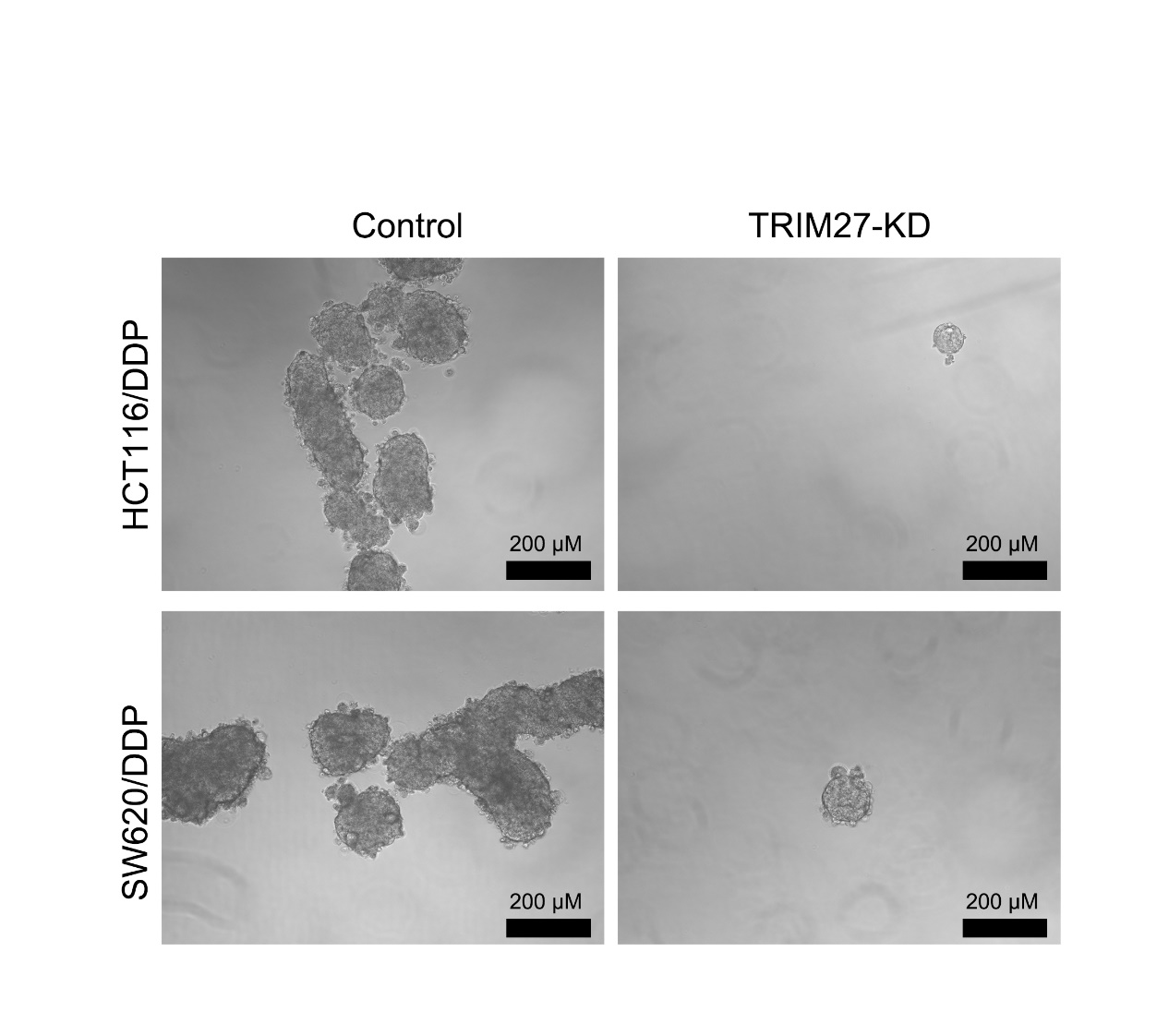
Figure S1** Representative figure of Spheroids formation and quantification from control or TRIM27-KD in HCT116/DDP and SW620/DDP cells. Scale bar, 200Μm.
